# Reduced Heme Levels Underlie the Exponential Growth Defect of the *Shewanella oneidensis hfq* Mutant

**DOI:** 10.1371/journal.pone.0109879

**Published:** 2014-10-30

**Authors:** Christopher M. Brennan, Nicholas Q. Mazzucca, Taylor Mezoian, Taylor M. Hunt, Meaghan L. Keane, Jessica N. Leonard, Shelby E. Scola, Emma N. Beer, Sarah Perdue, Brett J. Pellock

**Affiliations:** 1 Department of Biology, Providence College, Providence, Rhode Island, United States of America; 2 Department of Chemistry and Biochemistry, Providence College, Providence, Rhode Island, United States of America; East Carolina University School of Medicine, United States of America

## Abstract

The RNA chaperone Hfq fulfills important roles in small regulatory RNA (sRNA) function in many bacteria. Loss of Hfq in the dissimilatory metal reducing bacterium *Shewanella oneidensis* strain MR-1 results in slow exponential phase growth and a reduced terminal cell density at stationary phase. We have found that the exponential phase growth defect of the *hfq* mutant in LB is the result of reduced heme levels. Both heme levels and exponential phase growth of the *hfq* mutant can be completely restored by supplementing LB medium with 5-aminolevulinic acid (5-ALA), the first committed intermediate synthesized during heme synthesis. Increasing expression of *gtrA*, which encodes the enzyme that catalyzes the first step in heme biosynthesis, also restores heme levels and exponential phase growth of the *hfq* mutant. Taken together, our data indicate that reduced heme levels are responsible for the exponential growth defect of the *S. oneidensis hfq* mutant in LB medium and suggest that the *S. oneidensis hfq* mutant is deficient in heme production at the 5-ALA synthesis step.

## Introduction

The RNA chaperone Hfq is a highly conserved protein that mediates interactions between many regulatory sRNA molecules and their mRNA targets in bacteria (for reviews see [1]–[4]). Hfq protein monomers form a homohexameric ring capable of binding both regulatory small, noncoding RNAs (sRNAs) and their target mRNAs [5], [6]. These Hfq-RNA interactions stabilize sRNAs and help the sRNAs locate and interact with their mRNA targets. Because sRNA expression and subsequent changes in target gene expression help bacteria adapt to changing environmental conditions, Hfq plays key roles in regulating a wide variety of cellular functions.

Genetic loss-of-function studies have been widely employed to elucidate how Hfq functions to help regulate bacterial gene expression networks (for example see [7]–[10]). Consistent with a role in a multitude of cellular processes, loss of Hfq is typically pleiotropic, and *hfq* mutants exhibit a diverse array of bacterium-specific phenotypes. This suggests that, despite its high level of conservation, Hfq has evolved distinct roles in closely related bacteria. However, a common theme in the study of Hfq is that *hfq* mutants are typically less efficient at responding to the challenges of growth and stress conditions, which is consistent with the role of Hfq as a functional mediator for adaptive sRNAs. Though reduced sRNA function likely contributes to many *hfq* mutant phenotypes, the mechanisms by which loss of Hfq results in particular mutant phenotypes often remain obscure.

A null mutant of *hfq* in the dissimilatory metal-reducing bacterium *Shewanella oneidensis* was recently characterized [10]. Loss of *hfq* in *S. oneidensis* results in slow exponential phase growth, reduced stationary phase culture density, slow anaerobic growth, a reduced capacity for chromium reduction, and a greatly increased sensitivity to oxidative stress. *S. oneidensis* is of particular interest because anaerobically growing cells are capable of transferring electrons to a wide variety of extracellular terminal electron acceptors, including many soluble and insoluble metals [11], [12]. This capability has sparked interest in *S. oneidensis* as a potential bioremediating organism as well as an organism capable of producing electrical current in microbial fuel cells [13], [14]. Because bacteria in nature likely experience frequent metabolic changes, understanding how Hfq and sRNAs mediate adaptive cellular processes will provide useful insights into the genetic and physiological control mechanisms of *S. oneidensis*.

Heme molecules participate in a variety of important cellular processes. For example, heme is a key prosthetic group in cytochrome proteins used in electron transport chains. Heme also has other important physiological roles in iron storage and oxidative stress resistance. The importance of heme in bacterial growth is underscored by the fact that bacteria that are unable to synthesize heme, including many pathogens, must acquire it from their external environments [15]. Heme availability is particularly important for electron transport in *S. oneidensis*, which can potentially encode 42 different c-type cytochromes that are thought to confer the ability to use a diverse array of terminal electron acceptors [16], [17]. The predominant form of heme in *S. oneidensis* is heme C [18], which is covalently attached to two cysteine residues in cytochrome proteins by the activity of the heme lyase protein CcmF [19].

Heme biosynthesis is a well-conserved process (for reviews see [15], [20]). The first committed step in heme synthesis is the production of 5-aminolevulinic acid (5-ALA). In mammalian cells, yeast, and the α-proteobacteria, the HemA protein (5-ALA synthase) catalyzes the synthesis of 5-ALA in a single step from glycine and succinyl-CoA (the C_4_ pathway). In other organisms (the remaining eubacteria, plants, Archaea, and algae), 5-ALA is produced in a two step process (the C_5_ pathway). First, glutamyl tRNA reductase (GtrA/HemA) uses NADPH as a cofactor to reduce glutamate supplied by a charged glutamyl tRNA to a glutamate-1-semialdehyde (GSA) intermediate. GSA is then converted to 5-ALA by the enzyme GSA aminotransferase (HemL). Following 5-ALA synthesis, heme is produced using seven additional conserved enzyme-catalyzed reactions.

Regulation of heme biosynthesis in bacteria is largely focused on the activity of the first enzyme in the heme biosynthetic pathway (reviewed in [20]). Though bacteria modulate production of GtrA/HemA on the transcriptional level, this regulation is generally modest. In contrast, bacteria appear to exert a much larger influence on glutamyl tRNA reductase activity by posttranlationally regulating GtrA/HemA protein stability. For example, in *Salmonella typhimurium*, heme binds to the GtrA/HemA protein and promotes its proteolytic degradation via the Lon and ClpAP proteases [21]. Thus, high heme levels negatively regulate the first committed step in heme biosynthesis via anabolite repression.

In this study, we report the serendipitous discovery that reduced heme levels underlie the exponential growth phase defect of the *S. oneidensis hfq* mutant. Restoring heme levels in the *hfq* mutant by nutritional supplementation with 5-aminolevulinic acid or by exogenous production of the *S. oneidensis* glutamyl tRNA reductase (hereafter referred to as *gtrA* or GtrA to reflect the naming convention proposed by Panek and O'Brian [15]), the first enzyme in the heme biosynthesis pathway, restores exponential growth to wild type levels. Thus, the slow growth of the *S. oneidensis hfq* mutant in LB is due to reduced heme levels and not a combination of pleiotropic factors. In addition, the defect in heme synthesis in the *S. oneidensis hfq* mutant occurs at or before the 5-ALA synthesis step. Our findings represent an important advance in understanding the link between the *S. oneidensis hfq* mutant slow growth phenotype and a defect in a specific metabolic pathway.

## Results

### Growth on blood agar substantially rescues the small colony defect of the *S. oneidensis hfq* mutant

**Figure 1:**
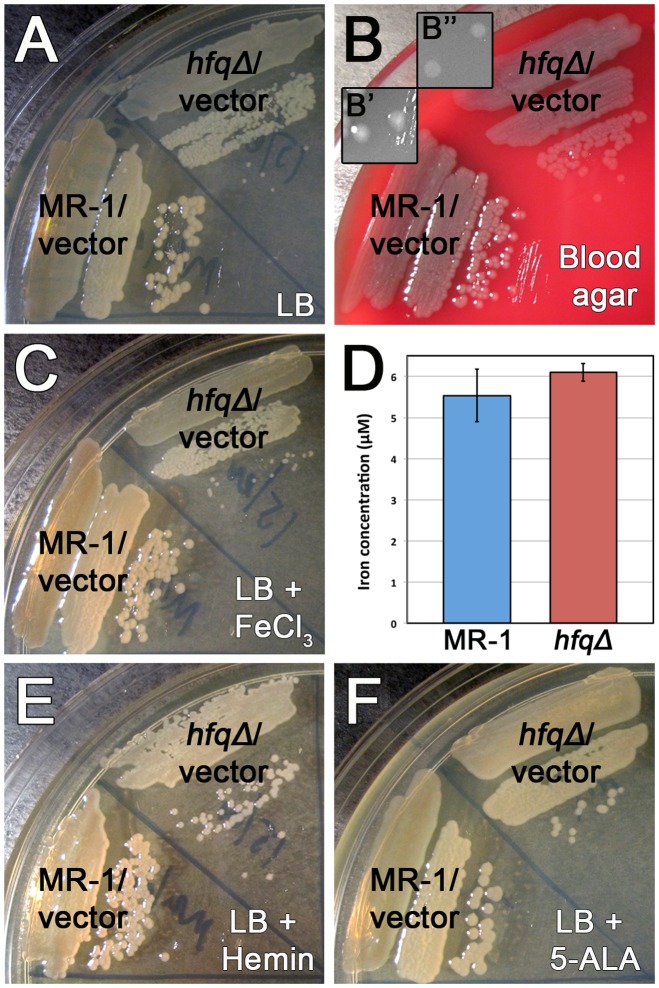
Heme or 5-ALA supplementation substantially rescues the colony size phenotype of the *hfq* mutant. Colony size comparisons of MR-1/pBBR1-MCS2 (vector), and *hfqΔ*/pBBR1-MCS2 (vector) streaked to single colonies on (A) LB Km, (B) TSA containing 5% sheep blood [including higher magnification insets for colony size comparison: (B′) MR-1/pBBR1-MCS2 (vector) colonies and (B″) *hfqΔ*/pBBR1-MCS2 (vector) colonies], (C) LB Km supplemented with 50 µM FeCl_3_, (E) LB Km supplemented with 50 µM hemin, or (F) LB Km supplemented with 50 µM 5-aminolevulinic acid (5-ALA). Plates were photographed following 23–25 hours of growth at 30°C. (D) Quantification of total free iron in wild type MR-1 and *hfqΔ* mutant cells using the ferrozine reagent. Concentration of detectable free iron was computed as described in Materials and Methods. Data presented is the mean of three independent cultures. Error bars indicate standard deviations. The difference between iron levels in MR-1 and the *hfqΔ* mutant is not statistically significant (P = 0.22 in an unpaired two-tailed Student's t-test).

Colonies formed by an *S. oneidensis hfq* mutant on LB plates are substantially smaller than colonies formed by cells containing a wild type copy of *hfq* (Figure 1A, [10]). Because the *hfq* mutant is highly sensitive to peroxide stress, we tested trypticase soy agar (TSA) medium containing 5% sheep blood as a potential qualitative measure to assay whether the *hfq* mutant produces higher levels of reactive oxygen species. Though the *hfq* mutant did not produce more heme oxidation (α hemolysis) than wild type cells, we were surprised to observe that growth on sheep blood agar substantially rescued the colony size defect of the *hfq* mutant (Figure 1B). The *hfq* mutant small colony defect on TSA without blood (Figure S1A) was comparable to the phenotype observed on LB (Figure 1A).

### Heme or 5-aminolevulinic acid substantially rescues the small colony phenotype of the *S. oneidensis hfq* mutant

Because most bacteria have the capacity to scavenge iron from hemoglobin to support vital cellular functions [27], we determined whether the addition of iron to the medium at concentrations approximating those found in trypticase soy agar plates containing 5% sheep blood could rescue the growth defect of the *hfq* mutant. Addition of 50 µM FeCl_3_ to LB plates, which already contain ∼17 µM iron [28], did not substantially alter the size of the *hfq* mutant colonies or the wild type colonies (Figure 1C), suggesting that iron is not the factor present in blood that rescues growth of the *hfq* mutant. An alternative possibility is that the *hfq* mutant might be defective in iron uptake, but not heme uptake, allowing the cells to scavenge iron by importing heme. However, exponentially growing *hfq* mutant and wild type cells contained similar levels of free iron per cell (Figure 1D). In addition, for both the MR-1 wild type and *hfq* mutant cultures we observed the same pattern of free iron levels. At lower cell densities, iron levels per cell are highest, but after culture density reaches an ABS_600_ of ∼4.0, the free iron content of the cells decreases (Figure S2C). Taken together, these data suggest that rescue of the *hfq* mutant small colony phenotype is due to a factor other than iron.

Since *hfq* mutant colonies are noticeably less pigmented than colonies of strains with a wild type copy of *hfq* (Figure 1A), and because the pink hue of *Shewanella* is largely the result of reduced heme in cytochrome proteins [29], [30], we hypothesized that growth rescue of the *hfq* mutant was due to the heme present in mammalian blood. Addition of 50 µM heme in the form of purified porcine hemin substantially rescued the small colony phenotype and partially restored the pink color of the *hfq* mutant (Figure 1E). This suggests that heme, but not iron alone, rescues the growth of the *hfq* mutant.

Synthesis of 5-aminolevulinic acid (5-ALA) is the first committed step in heme biosynthesis. 5-ALA synthesis is a critical focal point for regulation of heme biosynthesis, since 5-ALA is used exclusively for heme production [20]. Because exogenously-supplied heme partially rescues the *hfq* mutant small colony phenotype, we hypothesized that a defect in 5-ALA synthesis, and thus heme biosynthesis, results in slow growth of the *hfq* mutant. Addition of 50 µM 5-ALA to LB plates rescued the colony size defect of the *hfq* mutant to a similar extent as addition of 50 µM heme (Figure 1F). Because eight 5-ALA molecules are used to synthesize one heme molecule, we also determined whether higher levels of 5-ALA would increase the degree of colony size rescue. However addition of 400 µM 5-ALA rescued *hfq* mutant growth to a similar extent as 50 µM 5-ALA (Figure S1B).

Nutritional rescue by 5-ALA of the growth defect of the *hfq* mutant suggests that loss of *hfq* creates a heme-specific growth phenotype. This model is supported by our observation that increasing the nutritional content of LB by doubling the concentrations of the tryptone and yeast extract components failed to rescue the *hfq* mutant growth defect (Figure S1C). Because glutamyl tRNA^Glu^ supplies the glutamate that is converted into 5-ALA, we also determined whether supplementing LB with a mixture of glutamate, arginine, and serine could rescue the *hfq* mutant growth defect. However, addition of these amino acids at the concentrations they are found in modified M1 medium (see Materials and Methods) did not rescue *hfq* mutant colony size beyond that seen on LB alone (Figure S1D). Taken together, our data suggest that the *hfq* mutant is defective in heme biosynthesis at the 5-ALA synthesis step.

### The *hfq* mutant contains reduced levels of heme

**Figure 2:**
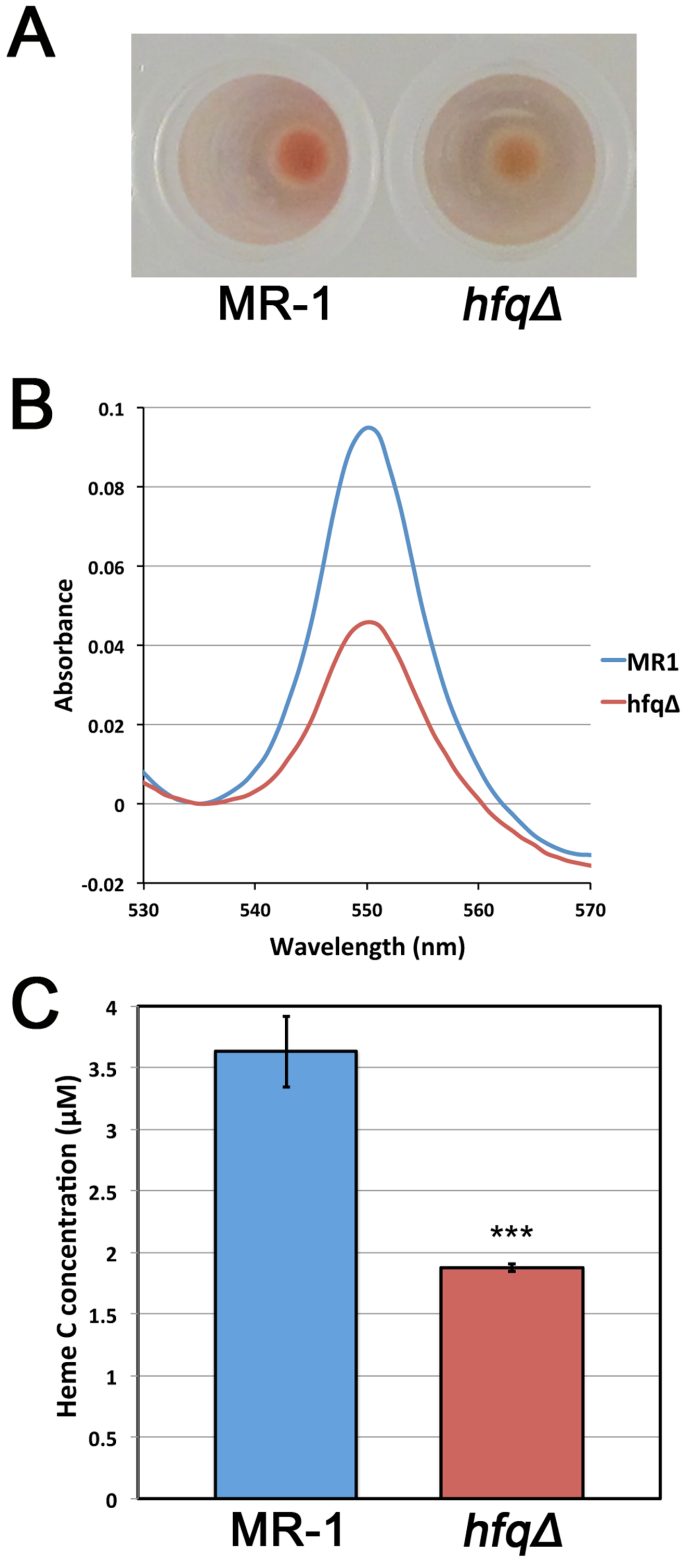
The *hfq* mutant is deficient in heme production. (A) Comparison of exponentially-growing MR-1 wild type and *hfqΔ* mutant cell pellet pigmentation. Both cell pellets are comprised of similar numbers of cells. (B) Superimposed heme assay subtraction spectra (reduced – oxidized) from single samples of exponentially-growing MR-1 wild type and *hfqΔ* mutant cultures. Data presented is typical of the difference observed for the two strains. (C) Quantification of heme concentrations from MR-1 wild type and *hfqΔ* mutant cultures. Concentration of detectable heme was computed as described in Materials and Methods. Data presented is the mean of three independent cultures. Error bars indicate standard deviations. *** indicate that the difference in heme levels between MR-1 and the *hfqΔ* mutant is statistically significant (P<0.001 in an unpaired two-tailed Student's t-test).

The pigmentation difference between wild type *S. oneidensis* colonies and *hfq* mutant colonies on LB plates (Figure 1A) is more striking when cell pellets of the two strains are compared (Figure 2A). To determine whether the reduced pigmentation of *hfq* mutant cells is in fact due to reduced levels of heme, we performed the pyridine hemochrome assay [23] on exponentially growing wild type MR-1 and *hfq* mutant cells. In this assay, the reduced form of heme C, the dominant heme species in *S. oneidensis* [18], produces an absorbance peak at 550 nm. The height of the 550 nm absorbance peak as measured from the absorbance trough at 535 nm is directly proportional to the amount of heme in the sample [23]. Analyses of the heme spectra from wild type and *hfq* mutant cultures (Figure 2B) and subsequent statistical analyses of the quantities of heme present in the samples (Figure 2C) indicated that exponentially growing wild type cells contain significantly more heme than *hfq* mutant cells.

### Increasing expression of *gtrA* rescues the small colony phenotype of the *S. oneidensis hfq* mutant

**Figure 3:**
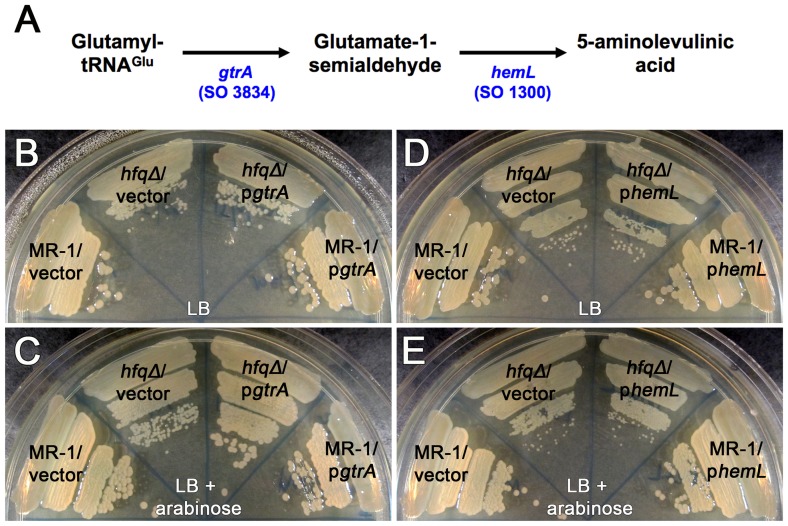
Exogenous *gtrA* expression rescues the colony size defect of the *hfq* mutant. (A) Putative two step pathway for the biosynthesis of 5-aminolevulinic acid in *Shewanella oneidensis* MR-1. (B and C) Colony size comparisons of MR-1/pBBAD-SP (vector), MR-1/pBBAD-*gtrA* (p*gtrA*), *hfqΔ*/pBBAD-SP (vector), and *hfqΔ*/pBBAD-*gtrA* (p*gtrA*), streaked to single colonies on (B) LB Km and (C) LB Km containing 0.005% arabinose. (D and E) Colony size comparisons of MR-1/pBBAD-SP (vector), MR-1/pBBAD-p*hemL* (p*hemL*), *hfqΔ*/pBBAD-SP (vector), and *hfqΔ*/pBBAD-p*hemL* (p*hemL*), on (D) LB Km and (E) LB Km containing 0.005% arabinose. Plates were photographed following 24 hours of growth at 30°C.

That addition of 5-ALA to the growth medium substantially rescued the small colony phenotype of the heme deficient *hfq* mutant suggests that a deficiency in heme production might be the result of a defect in 5-ALA synthesis. In *S. oneidensis*, 5-ALA is synthesized in a putative two-step process involving the genes *gtrA* and *hemL* (Figure 3A). GtrA protein converts glutamate donated by glutamyl tRNA^Glu^ into glutamate-1-semialdehyde, which is then converted into 5-ALA by the HemL protein. Since 5-ALA is used exclusively for heme production, we hypothesized that the heme biosynthesis defect of the *hfq* mutant is due to reduced function of the *gtrA* gene, the *hemL* gene, or both.

If lowered *gtrA* and/or *hemL* function underlies the heme biosynthesis defect of the *hfq* mutant, then increasing expression of *gtrA*, *hemL*, or both should rescue the mutant growth defect. To test this, we constructed arabinose-inducible plasmid expression vectors containing the *gtrA* gene, the *hemL* gene, or both the *gtrA* and *hemL* genes. We then constructed wild type and *hfq* mutant strains containing these plasmids and determined whether expression of *gtrA*, *hemL*, or both altered growth of these strains relative to wild type and *hfq* mutant strains containing the empty vector.

Exogenous expression of *gtrA* alone completely rescued the small colony phenotype of the *hfq* mutant (Figure 3B and 3C). In contrast, *hfq* mutant colonies expressing *hemL* alone were indistinguishable in size from *hfq* mutant colonies with vector alone (Figure 3D and 3E). Expressing both *gtrA* and *hemL* rescued *hfq* colony size to a similar extent as expressing *gtrA* alone (Figure S3). Taken together, our data suggest that reduced *gtrA* function underlies the colony size defect of the *hfq* mutant.

### Increasing heme availability via nutritional supplementation or genetic manipulation rescues the exponential growth defect of the *hfq* mutant

The small colony phenotype of the *hfq* mutant could be due to one or more *S. oneidensis hfq* mutant phenotypes, including the defect in exponential phase growth and/or saturation at a lower terminal density [10]. To determine how nutritional supplementation and genetic manipulation of the heme biosynthetic pathway influence culture growth kinetics and increase the size of *hfq* mutant colonies, we performed detailed analyses of both the growth kinetics and heme content of wild type and *hfq* mutant cultures in which we manipulated heme availability via nutritional or genetic means.

**Figure 4:**
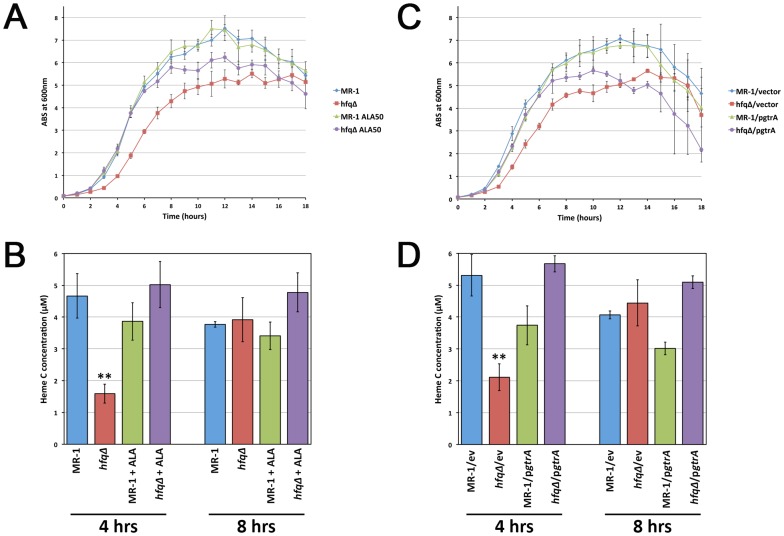
Restoring heme biosynthesis rescues the exponential phase growth defect of the *hfq* mutant. (A) Growth curves for MR-1/pBBAD-SP (MR-1) and *hfqΔ*/pBBAD-SP (*hfqΔ*) grown in either LB Km liquid medium or in LB Km liquid medium supplemented with 50 µM 5-ALA. (B) Heme content analysis of cells from MR-1/pBBAD-SP (MR-1) and *hfqΔ*/pBBAD-SP (*hfqΔ*) cultures grown for either 4 hours or 8 hours in either LB Km liquid medium or in LB Km liquid medium supplemented with 50 µM 5-ALA. (C) Growth curves for the wild type MR-1 strain and *hfqΔ* mutant strains containing either pBBAD-SP (vector/ev) or p*gtrA* grown in either LB Km or in LB Km containing 0.005% arabinose. (D) Heme content analysis of MR-1 and *hfqΔ* mutant cells containing either pBBAD-SP (vector/ev) or p*gtrA* grown in either LB Km or in LB Km containing 0.005% arabinose. Results presented for both growth curve and heme assays are the means from three independent cultures. Error bars in panels (A) and (C) indicate a 99% confidence interval (P = 0.01). Error bars in panels (B) and (D) indicate standard deviations. ** indicates that the difference between heme levels at 4 hours between MR1 and the *hfqΔ* mutant are statistically significant (P<0.0025 in an unpaired two-tailed Student's t-test).

Addition of 50 µM 5-ALA to LB liquid medium completely rescued the exponential phase growth defect of the *hfq* mutant (Figure 4A) and restored heme levels in the *hfq* mutant to wild type levels (Figure 4B). However, by 7–8 hours of culture growth, when heme levels in the unsupplemented *hfq* mutant were indistinguishable from those in wild type cells (Figure 4B and 4D), absorbance values for the *hfq* mutant plus 5-ALA stopped increasing and were no longer coincident with the MR-1 cultures (Figure 4A). The terminal density of stationary phase *hfq* mutant cultures was significantly increased by addition of 5-ALA, but these cultures never achieved the terminal density of wild type cultures (Figure 4A). This suggests that factors independent of heme levels are at least partially responsible for the reduced terminal density of the *hfq* mutant.

Increasing *gtrA* expression using an inducible plasmid vector also completely rescued the *hfq* mutant exponential phase growth defect in LB liquid medium, as growth of the *hfq* mutant expressing *gtrA* was indistinguishable from growth of wild type cells expressing *gtrA* (Figure 4C). We consistently observed that expression of *gtrA* slightly slows the growth of both the *hfq* mutant and wild type cells, indicating that exogenous *gtrA* expression mildly compromises growth of *S. oneidensis* cultures. Expression of *gtrA* restored heme levels in exponentially growing *hfq* mutant cells to wild type levels (Figure 4D). Similar to nutritional rescue of *hfq* mutant growth, genetic rescue of growth waned between 7–8 hours (Figure 4C).

These data indicate that the exponential phase growth defect of the *hfq* mutant is strongly linked to heme levels 2–3 fold lower (see Figures 2C, 4B, and 4D) than those found in wild type cells. This heme deficiency persists only until cultures begin to enter stationary phase, at which point the heme levels in the mutant cells are equivalent to those found in wild type cells (Figure 4B and 4D). Increasing heme availability rescues exponential growth of the *S. oneidensis hfq* mutant in LB, indicating that slow growth is due to reduced heme levels. In addition, our genetic and nutritional data strongly suggest that the *S. oneidensis hfq* mutant's heme defect occurs at the 5-ALA synthesis step.

### The *hfq* mutant contains modestly reduced levels of *gtrA* mRNA

To begin to elucidate the mechanism by which Hfq regulates *gtrA* function, we analyzed relative *gtrA* mRNA levels in exponentially growing wild type and *hfq* mutant cells. In 4 hour old cultures, we observed a modest, but significant reduction in *gtrA* mRNA levels in the *hfq* mutant relative to the wild type strain. When *gtrA* expression levels were normalized to 16S rRNA levels, the *hfq* mutant contained 0.63 fold the quantity (P<0.00002) of *gtrA* mRNA detected in wild type cultures (Data S1). When we independently normalized *gtrA* expression levels to *recA* mRNA levels, we observed that the *hfq* mutant contained 0.69 fold the quantity (P<0.0014) of *gtrA* mRNA detected in wild type cultures (Data S1). Thus, our data suggest that loss of *hfq* results in reduced *gtrA* transcription, an increased *gtrA* mRNA turnover rate, or both.

## Discussion

Here we report that the exponential growth defect of the *S. oneidensis hfq* mutant in LB medium is due to reduced levels of heme during exponential phase growth. The lowered heme levels are likely due to a defect in the 5-ALA synthesis step, as addition of 5-ALA to the medium or increased *gtrA* expression completely restores growth, while increasing the nutrient pool available to the cells does not restore growth. The fact that growth in nutritionally supplemented solid medium was not as robust as rescue in nutritionally supplemented liquid medium could be the result of local depletion of resources surrounding colonies on solid medium. However, increasing supplement concentrations did not further increase growth rescue on solid medium, suggesting that the difference in degree of growth rescue between solid and liquid medium is likely a reflection of differences in growth in the two media types.

That the *hfq* mutant exponential growth phenotype in LB is due to a deficiency in a single metabolic pathway is striking because loss of Hfq compromises many cellular processes. *S. oneidensis hfq* mutant cells growing in LB are partially auxotrophic for heme during exponential growth. Any other deficiencies that might influence exponential growth of the *hfq* mutant are not apparent when grown in LB liquid. A heme deficiency also contributes to the slow growth of the *hfq* mutant in medium other than LB, since heme supplementation of modified M1, a less rich defined medium, also substantially rescues the *hfq* colony size defect (Figure S4A and S4B). However in M1 medium, we observe a more modest rescue of *hfq* mutant growth with 5-ALA supplementation than we observe in LB (Figure S4C). This suggests that the *hfq* mutant has a reduced ability to convert 5-ALA into heme when it is grown on modified M1 medium. Indeed, supplementation of M1 medium with 400 µM ALA inhibits growth of the *hfq* mutant, possibly as the result of buildup of heme biosynthetic intermediates, some of which are toxic [31]. Thus, growth in less nutritionally rich medium appears to expose additional metabolic deficiencies of the *hfq* mutant.

Once *hfq* mutant cells reach the end of exponential phase, their heme levels are similar to those observed in similarly aged wild type cells. Thus, a heme deficiency cannot fully explain other *hfq* mutant phenotypes, such as the reduced stationary phase density or the late stationary phase survival defect of *hfq* mutant cultures. However, the terminal densities of *hfq* mutant cultures in which the heme defect has been rescued are significantly higher than the terminal densities of *hfq* mutant cultures alone. This indicates that the terminal density of a culture is in part, but not wholly, determined by the extent of its exponential phase growth. Further investigations into the mechanisms by which Hfq regulates heme levels should provide insight into why the *hfq* mutant has lower heme levels during exponential phase growth but not at the beginning of stationary phase.

An attractive model to explain slow exponential phase growth in the *hfq* mutant is that lower heme levels reduce the electron transport capacity of the cells, since heme is the redox center of cytochrome proteins. Reduced cytochrome function and diminished electron transport could also account for the slower chromium reduction kinetics and reduced anaerobic growth of the *hfq* mutant. Low heme levels could explain other *S. oneidensis hfq* mutant phenotypes. For example, because heme is a key part of the active site of the hydrogen peroxide degrading enzyme catalase, reduced heme availability could compromise catalase activity, making the *hfq* mutant more sensitive to oxidative stress. Heme is also a key part of the iron storage protein bacterioferritin, raising the possibility that changes in heme availability during exponential phase could impact iron homeostasis in the *hfq* mutant. However, regardless of bacterioferritin function, the levels of free iron in both wild type and *hfq* mutant cells are similar, suggesting that iron homeostasis may be minimally affected by loss of Hfq under the conditions tested.

Though Hfq appears to regulate heme production at the 5-ALA synthesis step, the mechanism by which loss of *hfq* results in lowered GtrA activity and thus reduced heme levels is not yet clear. Considering the modest reduction in *gtrA* mRNA levels in the *hfq* mutant during exponential growth, Hfq could function to stimulate transcription of *gtrA* and/or promote *gtrA* mRNA stability. However, though there is a statistically significant difference in *gtrA* mRNA levels between wild type and *hfq* mutant cells, it is not clear whether a ∼1.5 fold difference in *gtrA* expression is biologically significant in regard to heme levels. This raises the possibility that *S. oneidensis* regulates *gtrA* activity posttranscriptionally. For example, Hfq could promote the function of an unidentified sRNA that positively regulates the translation of *gtrA* mRNA by releasing attenuation due to a secondary structure that blocks the putative ribosome binding site. It is also possible that loss of Hfq could result in an increased heme or GtrA turnover rate, leading to lower steady state heme levels in exponentially growing *hfq* mutant cells. It is clear from our analyses that the regulatory mechanisms controlling heme levels in the *hfq* mutant differ substantially from those in wild type cells. We are currently investigating the mechanism(s) by which Hfq regulates heme levels in *S. oneidensis*.

## Materials and Methods

### Growth media, strains, and culture conditions

Unless otherwise specified, cultures were grown in LB medium (10g/L tryptone, 5g/L yeast extract, 10g/L NaCl) supplemented with kanamycin (Km). Other media used included modified M1 medium [10], [11], LB with double the normal quantities of tryptone and yeast extract (20g/L tryptone, 10g/L yeast extract, 10g/L NaCl), LB supplemented with 135.9 µM L-glutamic acid, 114.8 µM L-arginine, and 190.3 µM DL-serine, the concentrations of amino acids found in modified M1 medium [10], [11], trypticase soy agar (TSA), TSA supplemented with 5% sheep blood (Becton Dickinson), LB supplemented with 50 µM iron (in the form of FeSO_4_ or FeCl_3_), LB or M1 supplemented with 50 µM or 400 µM 5-aminolevulinic acid, and LB or M1 supplemented with 50 µM porcine hemin.

*S. oneidensis* strains used in this study are wild type strain MR-1 [11] and the MR-1 *hfqΔ* mutant [10] and their derivatives. *S. oneidensis* cultures and cultures containing both *E. coli* and *S. oneidensis* were grown at 30°C, while *E. coli* cultures were grown at 37°C. Antibiotics were used at the following concentrations: kanamycin (Km), 25 µg/mL; gentamicin (Gm), 5 µg/mL.

Bacteria grown on plates were streaked to single colonies in four phases from frozen permanent stocks or from colonies on streak plates that had been inoculated from frozen permanent stocks and grown overnight. For growth curves, overnight aerobic 5 mL LB Km cultures of *S. oneidensis* strains inoculated from frozen permanent stocks were diluted in LB Km to an ABS_600_ ≅0.1 and outgrown aerobically to mid exponential phase (ABS_600_ ≅0.4–1.0). These log phase cultures were then diluted to an ABS_600_ ≅0.1 in LB Km and grown aerobically in 125 mL Erlenmeyer flasks shaken at 250RPM. Culture turbidity (ABS_600_) was measured at regular intervals.

### Detection of free intracellular iron

Free, intracellular iron was detected via a modified version of a previously described protocol [22]. Overnight LB Km cultures of *S. oneidensis* strains were diluted to an ABS_600_ ≅0.1 and outgrown in LB Km. The equivalent of 5 mL of culture at an ABS_600_ of 1.0 was harvested for each strain after 4 hours of growth. Cells were pelleted in Eppendorf tubes and washed once by resuspending in sterile 0.85% (w/v) saline. The washed pellets were then resuspended in 1 mL of Bacterial Protein Extraction Reagent (B-PER, Thermo Scientific) containing 100 µg/mL lysozyme and incubated at room temperature for 15 minutes with regular mixing. 100 µL of 10 mM ferrozine reagent [3-(2-pyridyl)-5,6-diphenyl-1,2,4-triazine-4′, 4″-disulfonic acid sodium salt] (Sigma Aldrich) in 0.1 M ammonium acetate was added to each sample and mixed. 800 µL of this solution was then mixed with 150 µL of 1.4 M hydroxylamine hydrochloride in a fresh Eppendorf tube and incubated for 15 minutes at room temperature to reduce Fe(III) to Fe(II). The solution was then neutralized by adding 50 µL of 10 M ammonium acetate, pH 9.5. Samples were incubated for at least 4 hours at room temperature to allow full color development. Each sample was then pelleted for 2 minutes at maximum speed in a microcentrifuge. 600 µL of the supernatant was then transferred to a cuvette for ABS_562_ measurement in a spectrophotometer zeroed using a sample with assay reagents only. This assay is quantitative, as there is a linear relationship between iron concentration and ABS_562_ values (Figure S2A). Hemin alone did not produce a signal using this protocol (Figure S2B).

### Detection of total intracellular heme

Bacterial heme was detected via a modified version of a previously described protocol [23]. Overnight LB Km cultures of *S. oneidensis* strains were diluted to an ABS_600_ ≅0.1 and outgrown in LB Km. The equivalent of 10 mL of culture at an ABS_600_ of 1.0 was harvested in duplicate for each strain at the indicated time points. Cells were pelleted in Eppendorf tubes and washed once by resuspending in sterile 0.85% (w/v) saline. The washed pellets were then resuspended in 840 µL of Bacterial Protein Extraction Reagent (B-PER, Thermo Scientific) containing 100 µg/mL lysozyme and incubated at room temperature for 15 minutes with regular mixing. 200 µL of pyridine and 100 µL of 1.0 M NaOH was then mixed with each sample. One paired sample was oxidized by addition of 10 µL of 1 M potassium ferricyanide. The second paired sample was reduced by addition of 2–5 mg of sodium hydrosulfite powder. After zeroing the spectrophotometer with a sample with assay reagents only, an absorbance spectrum for each sample at 400–700 nm was obtained. A subtraction spectrum for each sample pair was generated by subtracting the oxidized spectrum from the reduced spectrum. Heme concentration was calculated using the absorbance difference between the peak at 550 nm and the trough at 535 nm from the subtraction spectrum and the millimolar extinction coefficient for heme C of 23.97 [23].

### Construction of plasmid vectors and arabinose induction

pBBAD-SP is an arabinose-inducible expression vector derived from the Km^r^ broad host range vector pBBR1-MCS2 [24]. A ∼1.7 kb fragment containing the *araC* gene, the P_BAD_ promoter, the multiple cloning site, and the transcriptional terminators from pBAD18 [25] was PCR amplified using the primers TCCGAGATCTTTATGACAACTTGACGGCTACATC and AGCGCTCGAGAACAAAAGAGTTTGTAGAAACGCAAAAAGG. This PCR fragment was restricted with both *Bgl*II and *Xho*I and then ligated to *Bam*HI and *Xho*I-restricted pBBR1-MCS2.

To construct plasmid vectors expressing *gtrA* (So_3834) alone, *hemL* (So_1300) alone, or both *gtrA* and *hemL* from *S. oneidensis* under control of an arabinose-inducible promoter, the appropriate open reading frame(s) was/were PCR amplified and cloned into pBBAD-SP. To construct pBBAD-*gtrA*, a *gtrA* PCR product generated using the 5′ primer GGCGAATTCCATAGGGCCCTAAGGAGGAAAAAAAATGAGCCTTGTAGCAATC and the 3′ primer GGCAAGCTTCCTTCATTTAACTCGCTAACC was digested with *Eco*RI and *Hin*dIII and ligated to *Eco*RI and *Hin*dIII-restricted pBBAD-SP. To construct pBBAD-*hemL*, a *hemL* PCR product generated using the 5′ primer GGCGAATTCCATAGGGCCCTAAGGAGGAAAAAAAATGACCCGTTCCGAAGC and the 3′ primer GGCAAGCTTGTAAATACTTAGTTTGCCGC was digested with *Eco*RI and *Hin*dIII and ligated to *Eco*RI and *Hin*dIII-restricted pBBAD-SP. To construct pBBAD-*gtrA+hemL*, a *gtrA* PCR product was generated using the 5′ primer GGCGAATTCCATAGGGCCCTAAGGAGGAAAAAAAATGAGCCTTGTAGCAATC and the 3′ primer GGCGAATTCCCTTCATTTAACTCGCTAACC, while a *hemL* PCR product was generated using the 5′ primer GGCGAATTCCATAGGGCCCTAAGGAGGAAAAAAAATGACCCGTTCCGAAGC and the 3′ primer GGCAAGCTTGTAAATACTTAGTTTGCCGC. The *gtrA* PCR product was digested with *Eco*RI, while the *hemL* PCR product was digested with both *Eco*RI and *Hin*dIII. These two fragments were ligated to pBBAD-SP restricted with *Eco*RI and *Hin*dIII to generate an arabinose-inducible *gtrA-hemL* synthetic operon.

*S. oneidensis* strains (wild type MR-1 and *hfq* mutant) containing the plasmids described above were constructed by first transforming the plasmids into the *tra* ^+^ *E. coli* strain S17-1 λ*pir* [26]. The plasmids were then mobilized into *S. oneidensis* via conjugal transfer. MR-1 transconjugants were isolated from modified M1 plates [10] containing Km, while *hfq* mutant transconjugants were isolated from LB Km Gm plates.

Induction of heme biosynthesis genes cloned downstream of the P_BAD_ promoter was accomplished by addition of 0.005% (w/v) arabinose to the medium. This arabinose concentration was selected to optimize growth rescue and minimize negative effects of exogenous *gtrA* expression (see Figure 4C). The slight rescue of the *hfq* mutant growth phenotype by the pBBAD-*gtrA* plasmid (Figure 3B) and the significant rescue by the pBBAD-*gtrA*+*hemL* plasmid (Figure S3A) without arabinose present suggests that there is a low level of expression from the P_BAD_ promoter in *S. oneidensis* on LB medium in the absence of induction.

### Preparation of total RNA and QRT-PCR analysis

Aerobic, 5 mL LB Km cultures of *S. oneidensis* strains inoculated from frozen permanent stocks and grown overnight were diluted in LB Km to an ABS_600_ ≅0.1 and outgrown aerobically until harvest 4 hours later. Total RNA was prepared using the RNeasy Protect Bacterial RNA Kit (Qiagen) following manufacturer instructions.

cDNA reaction mixtures were prepared with the AffinityScript QPCR cDNA synthesis kit (Agilent Technologies) according to manufacturer's instructions. cDNA synthesis was primed using random oligonucleotide nonamers provided in the cDNA synthesis kit, and 100 ng of total RNA was used as the template for each 20 µL reverse transcription (RT) reaction. RT reactions were incubated at 25°C for 5′, 42°C for 15′, 55°C for 15′, 95°C for 5′, and then held at 4°C. Reactions were used immediately or stored at −20°C for later use.

Target-specific primers for QPCR reactions (Table S1) were selected using the PrimerQuest tool available on the Integrated DNA Technologies (IDT) web site. QPCR reactions were performed using the Brilliant II SYBR Green QPCR Master Mix reagent and the Mx3000P QPCR System (both from Agilent Technologies) as per manufacturer instructions. For each target, up to three different sets of QPCR primers were evaluated for quantitative amplification and amplification efficiency using four serial dilutions of cDNA samples spanning a 64 fold concentration range. For *gtrA* and *recA* primers, cDNA dilutions used were undiluted, 1/4, 1/16, and 1/64, while for 16S primers dilutions were 1/16, 1/64, 1/256, and 1/1024. Technical data for primer sets selected for QPCR analyses are contained in Data S1. All primers were used at a concentration of 600 nM.

Quantitative RT-PCR analyses were performed on three independent biological replicates. Three technical replicates were analyzed for each biological replicate. QPCR reactions for *recA* and *gtrA* were performed using 1/4 dilutions of cDNA, while 16S rRNA reactions were performed using 1/256 dilutions of the cDNA. Threshold cycle (Ct) values were determined as per the software defaults. *gtrA* data was individually normalized to either 16S rRNA levels or *recA* mRNA levels. All amplification data were efficiency corrected using the primer pair standard curve data found in Data S1 and Table S1, and relative target quantities were calculated as per the software specifications. Statistical analyses of QPCR data were performed using unpaired two-tailed Student's t-tests to compare the means of the technical replicate data for the three biological replicates. Technical data for the QPCR reactions is contained in Data S1. Despite the multiple physiological differences associated with loss of Hfq, both 16S rRNA and recA mRNA levels were similar between exponentially-growing wild type and hfq mutant cells for each of the biological replicates (Data S1).

## Supporting Information

Data S1 **Data from QPCR experiments and primer set efficiency analyses.**
(XLSX)

Data S2 **Data for** **Figure 1D** **. Raw and processed data for total free iron assays.**
(XLSX)

Data S3 **Data for** **Figure 2B and 2C** **. Raw and processed data for heme assays.**
(XLSX)

Data S4 **Data for** **Figure 4** **. Raw and processed data for growth curves and heme assays.**
(XLSX)

Data S5 **Data for Figure S2. Raw and processed data from iron assay standard curve and from total free iron assays.**
(XLSX)

Figure S1 **Growth of wild type and** ***hfq*** **mutant strains on different media.** Colony size comparisons of MR-1/pBBR1-MCS2 (vector) and *hfqΔ*/pBBR1-MCS2 (vector) streaked to single colonies on (A) trypticase soy agar (TSA), (B) LB Km supplemented with 400 µM 5-ALA, (C) LB Km containing double the normal concentrations of tryptone and yeast extract (2X LB), and (D) LB Km supplemented with DL-serine, L-glutamic acid, and L-arginine (LB + AAs - see Materials and Methods).
(TIF)

Figure S2 **Total iron detection: standard curve and cell culture trends.** (A) Standard curve generated from ferrozine assays performed using known concentrations of FeCl_3_. The blue trend line from a linear regression analysis with the y-intercept set at zero indicates that the assay is quantitative within the indicated concentration range (ABS_562_ = 0.0182 * [Fe] in the sample). For this data set the coefficient of determination (R^2^) = 0.99705. (B) Results of iron assays using samples containing either 5 µM FeSO_4_ or 5 µM hemin. Data is the mean of three independent samples. Error bars indicate standard deviation. **** indicates that the difference between iron detected is statistically significant (P<0.0001 in an unpaired two-tailed Student's t-test). (C) Plot of ferrozine assay results (ABS_562_ values) from iron assays versus culture densities (ABS_600_ values) at time of harvest. Data is pooled from multiple independent experiments using both MR-1 wild type cells (blue data points) and *hfq* mutant cells (red data points).
(TIF)

Figure S3 **Exogenous expression of both** ***gtrA*** **and** ***hemL*** **rescues the colony size defect of the** ***hfq*** **mutant.** Colony size comparisons of MR-1/pBBAD-SP (vector), MR-1/pBBAD-p*gtrA*+*hemL* (p*gtrA*+*hemL*), *hfqΔ*/pBBAD-SP (vector), and *hfqΔ*/pBBAD-p*gtrA*+*hemL* (p*gtrA*+*hemL*) streaked to single colonies on (A) LB Km and (B) LB Km containing 0.005% arabinose. Plates were photographed following 24 hours of growth at 30°C.
(TIF)

Figure S4 **Growth of wild type and** ***hfq*** **mutant strains on solid M1 medium supplemented with heme or 5-ALA.** Colony size comparisons of MR-1/pBBR1-MCS2 (vector) and *hfqΔ*/pBBR1-MCS2 (vector) streaked to single colonies on (A) M1 Km medium, (B) M1 Km medium supplemented with 50 µM hemin, (C) M1 Km medium supplemented with 50 µM 5-aminolevulinic acid (5-ALA), and (D) M1 Km medium supplemented with 400 µM 5-ALA. Plates were photographed following 72 hours of growth at 30°C.
(TIF)

Table S1 **Oligonucleotide primers used for QPCR analyses.**
(PDF)
