# Supplementary figures and images for: Reduced Heme Levels Underlie the Exponential Growth Defect of the Shewanella oneidensis hfq Mutant
Source: PLoS One. 2014 Oct 30;9(10):e109879. doi: 10.1371/journal.pone.0109879 (PMC4214671; doi:10.1371/journal.pone.0109879)

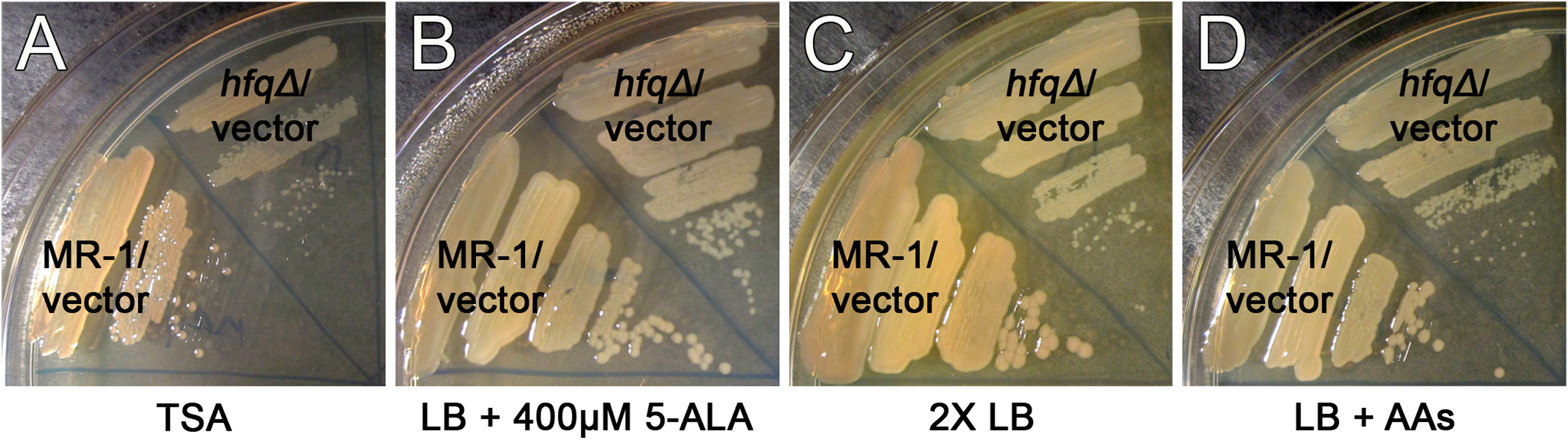

Supplement: Figure S1 — Growth of wild type and hfq mutant strains on different media. Colony size comparisons of MR-1/pBBR1-MCS2 (vector) and hfqΔ/pBBR1-MCS2 (vector) streaked to single colonies on (A) trypticase soy agar (TSA), (B) LB Km supplemented with 400 µM 5-ALA, (C) LB Km containing double the normal concentrations of tryptone and yeast extract (2X LB), and (D) LB Km supplemented with DL-serine, L-glutamic acid, and L-arginine (LB + AAs - see Materials and Methods). (TIF) [file pone.0109879.s006.tif]

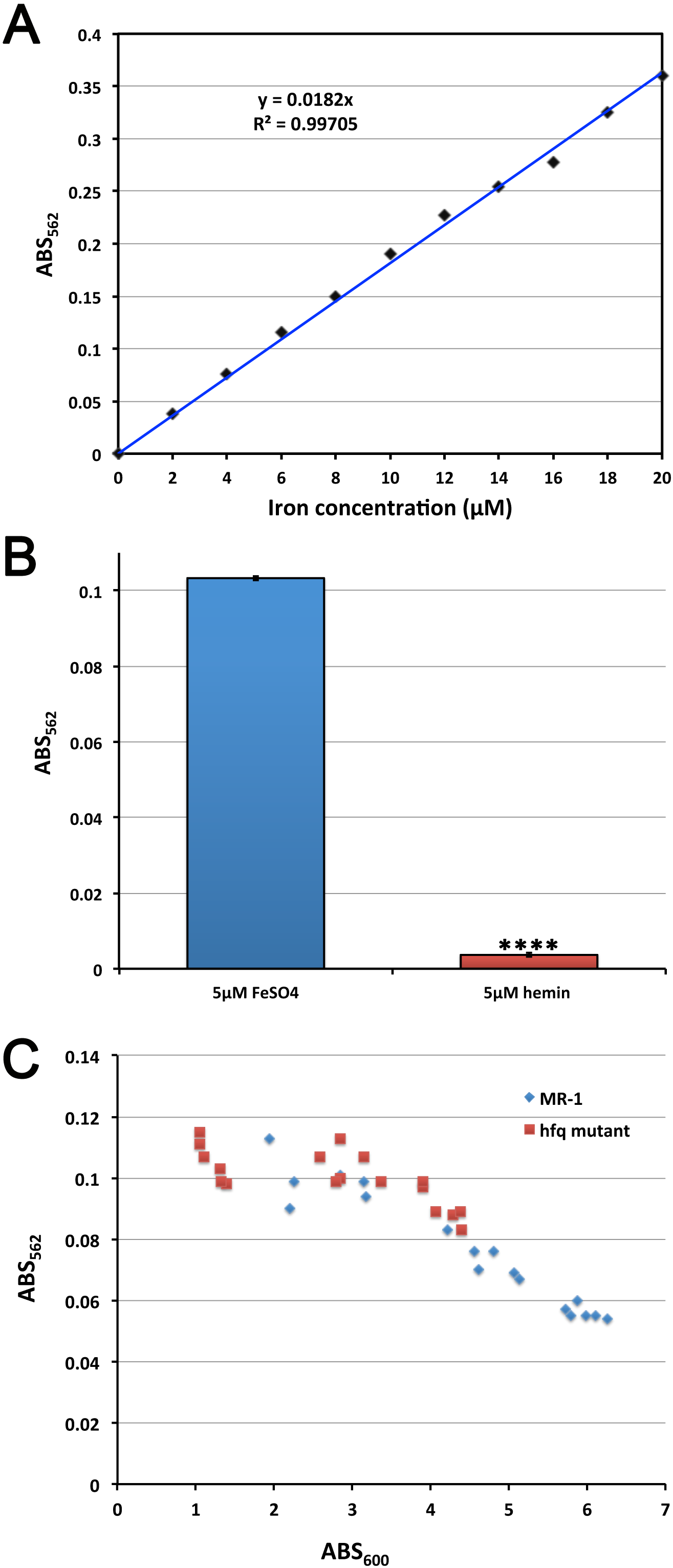

Supplement: Figure S2 — Total iron detection: standard curve and cell culture trends. (A) Standard curve generated from ferrozine assays performed using known concentrations of FeCl3. The blue trend line from a linear regression analysis with the y-intercept set at zero indicates that the assay is quantitative within the indicated concentration range (ABS562 = 0.0182 * [Fe] in the sample). For this data set the coefficient of determination (R2) = 0.99705. (B) Results of iron assays using samples containing either 5 µM FeSO4 or 5 µM hemin. Data is the mean of three independent samples. Error bars indicate standard deviation. **** indicates that the difference between iron detected is statistically significant (P<0.0001 in an unpaired two-tailed Student's t-test). (C) Plot of ferrozine assay results (ABS562 values) from iron assays versus culture densities (ABS600 values) at time of harvest. Data is pooled from multiple independent experiments using both MR-1 wild type cells (blue data points) and hfq mutant cells (red data points). (TIF) [file pone.0109879.s007.tif]

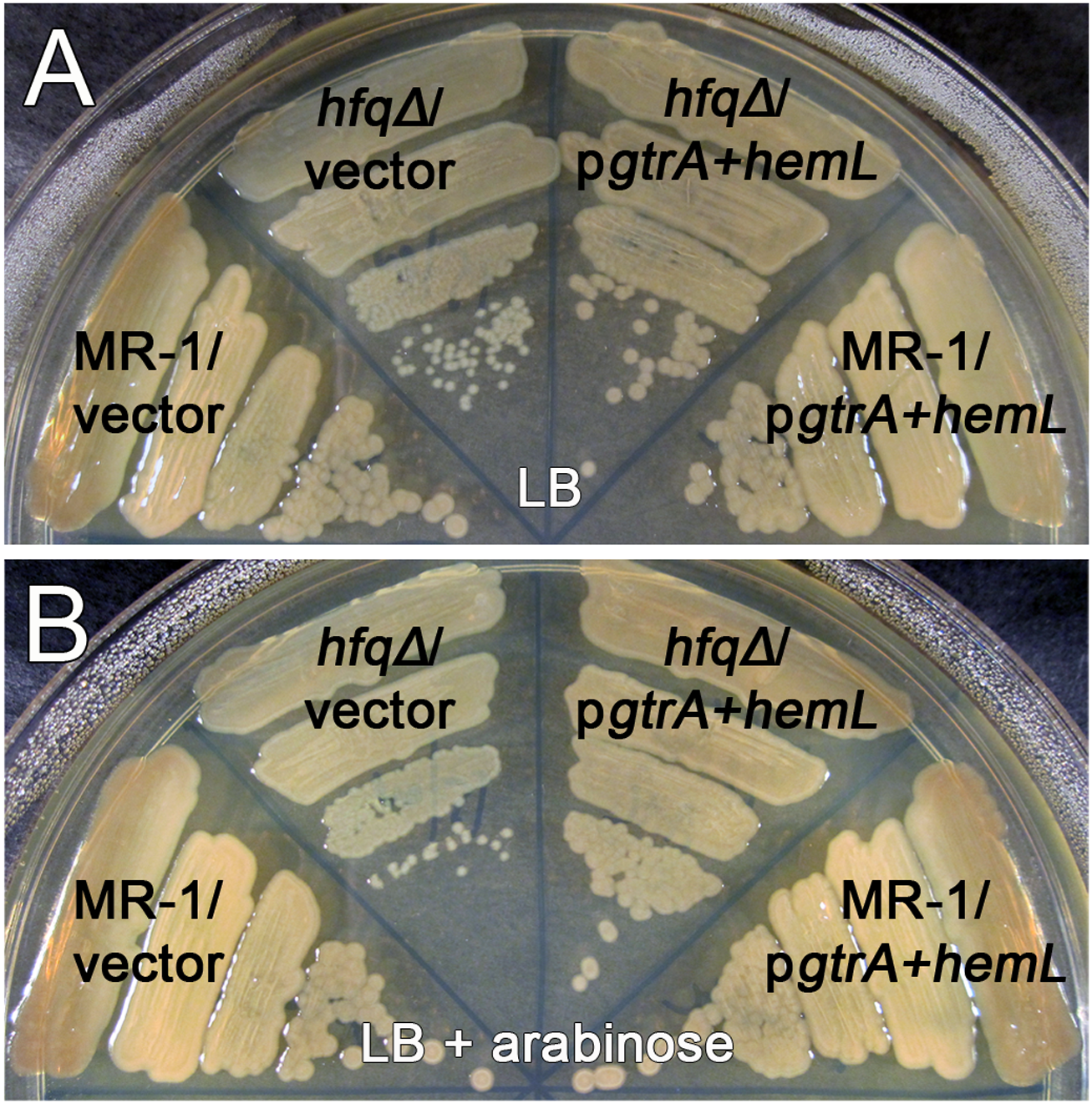

Supplement: Figure S3 — Exogenous expression of both gtrA and hemL rescues the colony size defect of the hfq mutant. Colony size comparisons of MR-1/pBBAD-SP (vector), MR-1/pBBAD-pgtrA+hemL (pgtrA+hemL), hfqΔ/pBBAD-SP (vector), and hfqΔ/pBBAD-pgtrA+hemL (pgtrA+hemL) streaked to single colonies on (A) LB Km and (B) LB Km containing 0.005% arabinose. Plates were photographed following 24 hours of growth at 30°C. (TIF) [file pone.0109879.s008.tif]

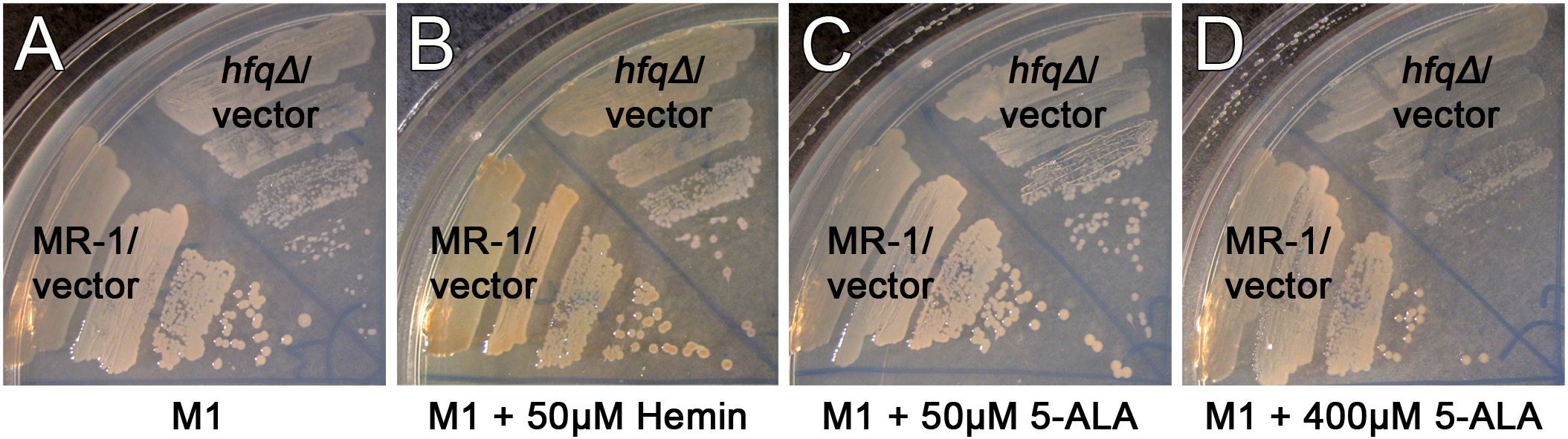

Supplement: Figure S4 — Growth of wild type and hfq mutant strains on solid M1 medium supplemented with heme or 5-ALA. Colony size comparisons of MR-1/pBBR1-MCS2 (vector) and hfqΔ/pBBR1-MCS2 (vector) streaked to single colonies on (A) M1 Km medium, (B) M1 Km medium supplemented with 50 µM hemin, (C) M1 Km medium supplemented with 50 µM 5-aminolevulinic acid (5-ALA), and (D) M1 Km medium supplemented with 400 µM 5-ALA. Plates were photographed following 72 hours of growth at 30°C. (TIF) [file pone.0109879.s009.tif]
