# Supplementary material for: Reduced Heme Levels Underlie the Exponential Growth Defect of the Shewanella oneidensis hfq Mutant
Source: PLoS One. 2014 Oct 30;9(10):e109879. doi: 10.1371/journal.pone.0109879 (PMC4214671; doi:10.1371/journal.pone.0109879)
Supplement: Table S1 — Oligonucleotide primers used for QPCR analyses. (PDF) [file pone.0109879.s010.pdf]

Table S1 – Oligonucleotide primers used for QPCR analyses

Primer name	Primer sequence	Primer pair efficiency
gtrA FWD Q1	CTTGGTAAAGGCATGGTAGAA	99.3%
gtrA REV Q1	GCGACTTCTGGCTCAATATC	
16S FWD Q3	CACAAAGCTCGTCGTAGTC	101%
16S REV Q3	GTATTCACCGTGGCATTCT	
recA FWD Q1	AGCAAGCCCTTGAGATTTG	101.5%
recA REV Q1	TCCGCCTTTGGTGTTAATG	
